# Development of Chronic Kidney Disease Screening Integrative Care Model Led by Community Pharmacists

**DOI:** 10.3390/pharmacy13010027

**Published:** 2025-02-14

**Authors:** Piangkwan Srimongkhol, Sirirat Anutrakulchai, Amponpun Theeranut, Nonglak Methakanjanasak, Sunee Lertsinudom

**Affiliations:** 1 College of Pharmacotherapy Thailand, Nonthaburi 11000, Thailand; 2 Division of Clinical Pharmacy, Faculty of Pharmacy, Mahasarakham University, Maha Sarakham 44150, Thailand; 3 Department of Internal Medicine, Faculty of Medicine, Khon Kaen University, Khon Kaen 40002, Thailand; sirirt_a@kku.ac.th; 4 Faculty of Nursing, Khon Kaen University, Khon Kaen 40002, Thailand; amptee@kku.ac.th (A.T.); nonchu@kku.ac.th (N.M.); 5 Division of Clinical Pharmacy, Faculty of Pharmaceutical Sciences, Khon Kaen University, Khon Kaen 40002, Thailand

**Keywords:** action research, community pharmacy, self-awareness, microalbuminuria, renal disease, screening program

## Abstract

Background: The prevalence of chronic kidney disease (CKD) is rising, increasing demand for renal replacement therapy (RRT). Community pharmacies, as accessible healthcare hubs, can play a pivotal role in CKD prevention. This study aimed to develop care models for community pharmacies to optimize medication use, encourage behavior modification, and promote self-management among at-risk individuals. Methods: Conducted between June 2017 and July 2018, this study utilized an action research approach. Microalbuminuria was assessed using urine dipsticks, and pharmacists applied behavioral change and self-management support (SMS) strategies to slow CKD progression. Participants were categorized by albuminuria levels and enrolled in pharmacist-led care programs, with follow-up assessments at weeks 0 and 12. Results: Of 521 participants screened, 57% tested positive for albuminuria. For these individuals, serum creatinine testing and referrals to primary care were initiated. Self-management behavior assessment (S1) scores significantly improved (*p* = 0.024). Key factors associated with urine albumin levels included age < 60 years (OR = 0.44), diabetes (OR = 3.69), hypertension (OR = 2.01), BMI < 27.5 kg/m^2^ (OR = 0.42), eGFR ≥ 60 mL/min/1.73 m^2^ (OR = 3.34), lower systolic (OR = 0.55) and diastolic blood pressure (OR = 0.34), and fasting plasma glucose < 126 mg/dL (OR = 0.29). Conclusions: Community pharmacist-led albuminuria screening effectively supports CKD prevention and enhances self-awareness within communities.

## 1. Introduction

Chronic kidney disease (CKD) is one of the most common noncommunicable chronic diseases, with a prevalence of 17.5% in Thailand [1]. The number of Thai CKD patients undergoing renal replacement therapy (RRT) increased from 58,385 in 2012 to 69,528 in 2013, 78,044 in 2014, and 85,848 in 2015 [2]. The three leading causes of CKD in Thailand are diabetes, hypertension, and urinary tract obstruction, which account for 38.57%, 30.71%, and 3.74% of cases, respectively [3].

A survey in Thailand found that more than one-third of the general population with diabetes was unaware of their diagnosis, while 44.7% of those with hypertension were unaware of their condition [4]. This lack of self-awareness increases the risk of developing complications, which is likely to escalate each year [5]. In 2017, the National Health Security Office (NHSO) proposed a concept for the control and prevention of the severity of diabetes and hypertension that included three components: (1) Primary prevention focuses on protecting the general population from disease by screening for risk factors, changing behavior, and providing appropriate knowledge and guidance. (2) Secondary prevention is the prevention of patients with diabetes and hypertension from developing renal and eye complications or stroke. It involves the necessary laboratory evaluations, supporting access to medications, and improving the quality of medical services. Both medication therapy and continuous drug monitoring are essential components of disease prevention. (3) Tertiary prevention focuses on the prevention of patients with complications caused by diabetes and hypertension, mortality, or disability by providing CKD clinics and renal replacement services to patients with end-stage renal disease [6].

Accredited community pharmacies in Thailand are part of the public health service system and can provide patient care services, including (1) screening for diabetes and hypertension, (2) improving the quality of medication management, and (3) participating in the modification of drug behavior and healthcare [7]. Accredited community pharmacies that participate in the National Health Insurance System offer comprehensive primary prevention services, such as chronic disease screening, smoking cessation, and medication therapy management (MTM). Additionally, a health prevention program (PP) is offered to patients in community pharmacies as well. These services demonstrate that accredited community pharmacies are easily accessible as screening resources for the public [6].

The current ESC and ACC guidelines emphasize the critical role of comprehensive kidney function assessment, which includes both serum creatinine-based estimated glomerular filtration rate (eGFR) and albuminuria evaluation across various aspects of cardiovascular risk management. The ESC 2021 Guidelines on Cardiovascular Disease Prevention recommend the routine measurement of the urine albumin-to-creatinine ratio (UACR) in hypertensive patients, highlighting its value for detecting early renal damage and refining cardiovascular risk stratification [8]. These guidelines also advocate for regular eGFR assessments as part of a holistic approach to identify and manage CKD.

Similarly, the ACC/AHA 2017 Guidelines on Hypertension recognize albuminuria as a key marker of target organ damage and support the use of both eGFR and UACR testing to guide treatment decisions and monitor disease progression [9]. The dual assessment of eGFR and UACR allows for a more accurate identification of CKD, enabling timely interventions to mitigate cardiovascular and renal complications.

Furthermore, the ESC 2019 Guidelines on Diabetes, Pre-Diabetes, and Cardiovascular Diseases underscore the importance of albuminuria screening in patients with diabetes as an indicator of elevated cardiovascular risk. These guidelines recommend regular assessments of both UACR and eGFR to facilitate the early detection of CKD, guide therapeutic strategies, and reduce the likelihood of complications [10].

The 2015 public health service guidelines for CKD patients prior to RRT recommend annual CKD screening for high-risk individuals to facilitate early diagnosis [11,12,13]. However, a kidney screening service is not yet available in accredited community pharmacies in Thailand presently. Therefore, this study aimed to (1) develop high-quality kidney screening protocols for accredited community pharmacies, (2) develop a protocol for transferring patients with chronic proteinuria to higher-level service units, and (3) manage medication use, modify behavior, and promote self-management to slow the progression of CKD in at-risk individuals or those with proteinuria.

## 2. Materials and Methods

### 2.1. Study Design

This study adopted an action research approach and consisted of three phases: phase 1—developing a practical care model; phase 2—care model implementation; and phase 3—evaluation. This study was approved by the Ethics Committee for Human Research (HE602167), Khon Kaen University, Thailand. Explicit informed consent was obtained from all participants.

### 2.2. Setting

This study was conducted in Mueang District, Khon Kaen Province, Thailand, in 2017. The district encompasses an area of 953.4 square kilometers and has a population of 382,156 [14].

### 2.3. Participants

#### 2.3.1. Participants in the Development and Implementation of the Program

The primary target population for this intervention was a network of 13 accredited community pharmacies in Khon Kaen municipality that were registered with the Khon Kaen Provincial Public Health Office.

#### 2.3.2. Participants in CKD Screening

The target population for this study was individuals aged at least 18 years who visited the pharmacy network of accredited community pharmacies and provided informed consent to participate.

Inclusion criteria:

(1) Age ≥ 18 years with at least one of the following: (1) diabetes, (2) hypertension, (3) systemic infections such as pyelonephritis or endocarditis, (4) cardiovascular disease, (5) recurrent upper urinary tract infections, (6) gout or elevated serum uric acid levels, (7) regular use of non-steroidal anti-inflammatory drugs (NSAIDs) or nephrotoxic medications, (8) decreased renal mass or unilateral kidney, congenital or acquired, (9) family history of CKD, (10) detected kidney stones or urinary tract stones, (11) three or more kidney cysts detected.
(2) Age ≥ 60 years without comorbidities.

Exclusion criteria:

(1) Blood creatinine test results obtained within one year prior to study participation, as documented in the patient’s medical record or laboratory certificate.
(2) Inability to communicate or hearing impairment without supervision, and autoimmune disease that can cause kidney disease.

### 2.4. Program

#### 2.4.1. Program Development

To develop the CKD care model, the content validity index (CVI) was calculated to ensure the validity of the instruments used in the study. A panel of five experts in pharmacy practice, public health, and behavioral sciences evaluated the content of the self-management behavior assessment (S1) and the self-care ability assessment (S2) forms. These experts were selected based on their extensive experience in instrument development and validation. A CVI evaluation form was provided to each expert. This form included criteria such as relevance, clarity, simplicity, and comprehensiveness of each item in the questionnaire. Experts rated each item on a 4-point Likert scale, where 1 = not relevant, 2 = somewhat relevant, 3 = quite relevant, and 4 = highly relevant. The item-level CVI (I-CVI) was calculated as the proportion of experts rating an item as either 3 or 4. The scale-level CVI (S-CVI) was derived by averaging the I-CVI scores across all items. Based on these evaluations, the CVI was 0.93 for the self-management behavior assessment (S1) and 1.00 for the self-care ability assessment (S2), indicating excellent content validity. Additionally, piloting was conducted in a subset of community pharmacies before the main study to assess the feasibility, usability, and clarity of the guidelines. Feedback from this pilot phase informed the final version of the model. Consistent terminology for the questionnaires and assessments is ensured throughout the manuscript to improve clarity and alignment.

Thereafter, we purposively selected 13 accredited community pharmacies. We first conducted a literature review to develop a preliminary screening model. We then brainstormed with selected pharmacists to develop guidelines for the screening, referral, behavior modification, and self-management of high-risk individuals. Finally, we presented the model to 13 pharmacists from 13 accredited community pharmacies to obtain feedback to improve the model for practical implementation.

**Figure 1:**
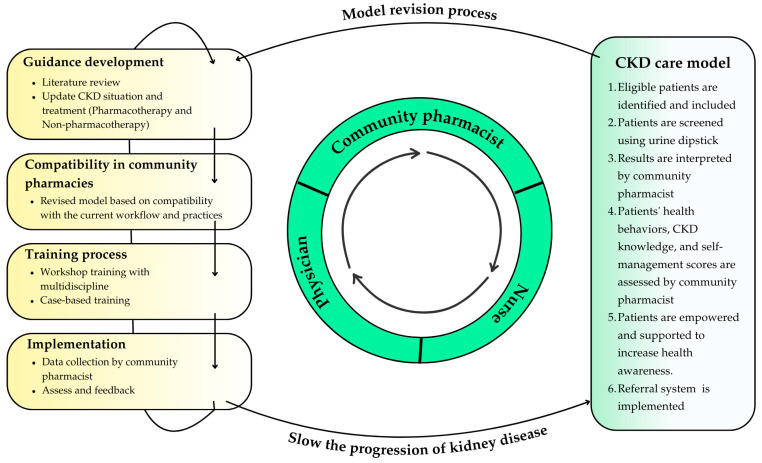
Model components and intervention in accredited community pharmacies.

Following the implementation of the model, the researchers convened a focus group meeting with pharmacists to reflect on the operational guidelines. The discussion covered the implementation of guidelines, tools used, problems encountered, factors influencing feasibility, practical and non-practical activities, and reasons for non-adherence to the plan. The goal of the meeting was to reach a consensus on how to improve the CKD screening program and develop a reusable care model. Model components and interventions were established based on the inputs and objectives of the stakeholders, and are shown in Figure 1 and Table 1.

#### 2.4.2. Program Implementation

The first CKD care model was implemented at accredited community pharmacies in July 2017. Pharmacists from accredited community pharmacies participated in a training workshop on the CKD screening program. Pharmacists who completed the training workshop activities and passed the assessment were certified to implement the model. Certified pharmacists conducted the care model on specific dates, scheduling screening, follow-up, and encouragement visits for the included participants. Pharmacists provided CKD screening programs daily during pharmacy operating hours. All at-risk individuals underwent a self-care assessment, received an education package, and took a follow-up albuminuria test at week 12. If urine results at week 0 were positive, the confirmed glomerular filtration rate (GFR) was required.

The pharmacists reviewed the patient’s self-management support (SMS) score, which was derived from a validated questionnaire designed to assess the patient’s self-management capabilities and knowledge regarding kidney disease. A standardized cutoff score was utilized to determine whether the patient required additional support to independently slow kidney disease progression. Specifically, patients scoring below 50% on the SMS questionnaire were categorized as needing further intervention. In addition to the SMS score, the pharmacists assessed the patient’s self-care ability using a separate self-care ability assessment form (S2). This form included a scoring system where a score of less than 5 indicated insufficient self-care abilities, prompting tailored educational or behavioral interventions. While the SMS score provided an overall measure of the patient’s self-management readiness, the self-care ability score offered a more specific evaluation of practical self-care capabilities. The two assessments were complementary, enabling pharmacists to identify patients who required additional support and customize their intervention strategies accordingly. In these cases, the pharmacist conducted an intensive follow-up every six weeks to address any identified problems and provided counseling. If further specialized examination was required, the patient was referred to a physician.

### 2.5. Outcomes

The model outcomes evaluated encompassed patients’ knowledge of CKD, as well as metrics derived from the SMS framework. Specifically, this included the assessment of self-management behaviors (S1) and self-care abilities (S2).

### 2.6. Data Collection

This study employed a three-part questionnaire to gather data, which included the following sections.

1. Demographic data:

Demographic information such as age, gender, level of education, CKD risks, medications, and urine albumin at baseline was collected by community pharmacists.

2. CKD knowledge questionnaire:

This 11-question assessment was used to evaluate participants’ knowledge of CKD. The scores are interpreted as follows: “high 8–11 points”, “medium 4–7 points”, and “low 0–3 points”. The questions were developed using guidelines from the Chronic Kidney Disease Outcomes Quality Initiative (KDOQI) and Kidney Disease Improving Global Outcomes (KDIGO), emphasizing fundamental knowledge areas such as kidney function, risk factors, and lifestyle modifications. Categories of “high,” “medium,” and “low” were based on the scoring thresholds determined during pilot testing, ensuring meaningful differentiation between knowledge levels.

3. The SMS form is divided into two parts:
  (1) The self-management behavior assessment (S1): This 11-item assessment was used to evaluate participants’ health behaviors. The scores are interpreted as follows: “good—35–44 points”, “fair—23–34 points”, “poor—11–22 points”. The categories were adapted from validated self-management frameworks that classify behaviors into “good,” “fair,” and “poor” based on participant responses. These thresholds were determined using prior research on self-care in chronic disease management and feedback from experts during the model development phase.
  (2) The self-care ability assessment form (S2): This was assessed at week 0 and 12. A score of 10 indicates that the participant takes good care of themselves, while a score of 1 indicates that the participant does not and needs thorough attention.

4. Instruments
  (1) Tools to educate and support self-management include a video and poster on CKD developed by CKDNET group.
  (2) A urine dipstick screening tool (Cobas Micral-Test^®^ version 11544039, Roche, Thailand) was used for microalbumin testing. Serum creatinine levels were measured using the Beckman Coulter LX20 PRO analyzer with the modified Jaffe method, and the results were used to calculate estimated glomerular filtration rate (eGFR) based on the CKD-EPI equation. The following guidelines were applied for patient follow-up and management:
    - If the urine dipstick test results were negative for albuminuria, patients were scheduled for follow-up testing at week 12.
    - If the urine dipstick test results were positive for albuminuria with an eGFR < 60 mL/min/1.73 m^2^, patients were followed up again at week 12 and referred to a primary care unit (PCU) for evaluation and treatment by a physician.
    - If the urine dipstick test results were positive for albuminuria with an eGFR ≥ 60 mL/min/1.73 m^2^, patients were scheduled for repeat testing at week 12.

### 2.7. Data Analysis

Demographic data were analyzed using descriptive statistics (means ± SDs or percentage) where appropriate. For outcomes that were compared pre- and post-intervention (average scores of behaviors, knowledge, and SMS), the Wilcoxon signed-rank test was used to analyze the data. The chi-square test was used to analyze categorical variables. Significance levels were set at 0.05.

To screen factors potentially associated with urine albumin level, a univariable analysis was conducted. Variables with a *p*-value < 0.25 were included in the subsequent binary logistic regression analysis [15]. The binary logistic regression model was then employed to assess the relationships between the identified factors and urine albumin levels. The results were interpreted using odds ratios (ORs) and their corresponding 95% confidence intervals (CIs), with statistical significance defined at a threshold of *p* < 0.05. Data were managed and analyzed using Stata version 14 software (StataCorp, College Station, TX, USA: Serial number: 401506248924).

In terms of sensitivity and specificity analysis, the reference standard for classifying patients into CKD and non-CKD groups was defined based on clinical guidelines. CKD was identified by the presence of albuminuria ≥ 30 mg/dL and/or a sustained eGFR < 60 mL/min/1.73 m^2^ across two measurements taken at least 12 weeks apart. Non-CKD was defined as the absence of albuminuria and an eGFR ≥ 60 mL/min/1.73 m^2^. These criteria were applied to classify participants before evaluating the diagnostic performance of the urine dipsticks.

Sensitivity, specificity, positive predictive value (PPV), and negative predictive value (NPV) of the urine dipstick screening tool were calculated by comparing the results of the dipstick tests to this reference standard. The effectiveness of a diagnostic test is determined by its sensitivity and specificity. There are four possible outcomes for a test [16]:

- True positive (TP) refers to individuals who have the disease and the test correctly identifies them as positive (accurate test result).
- False positive (FP) refers to individuals who do not have the disease, but the test incorrectly identifies them as positive (inaccurate test result).
- True negative (TN) refers to individuals who do not have the disease and the test correctly identifies them as negative (accurate test result).
- False negative (FN) refers to individuals who have the disease, but the test incorrectly identifies them as negative (inaccurate test result).

The sensitivity and specificity values can then be calculated using the following formulas:

(1) $$Sensitivity=\frac{True\;positive\;(TP)}{True\;positive\;(TP)+False\;negative\;(FN)}$$

(2) $$Specificity=\frac{True\;negative\;(TN)}{True\;negative\;(TN)+False\;positive\;(FP)}$$

## 3. Results

**Figure 2:**
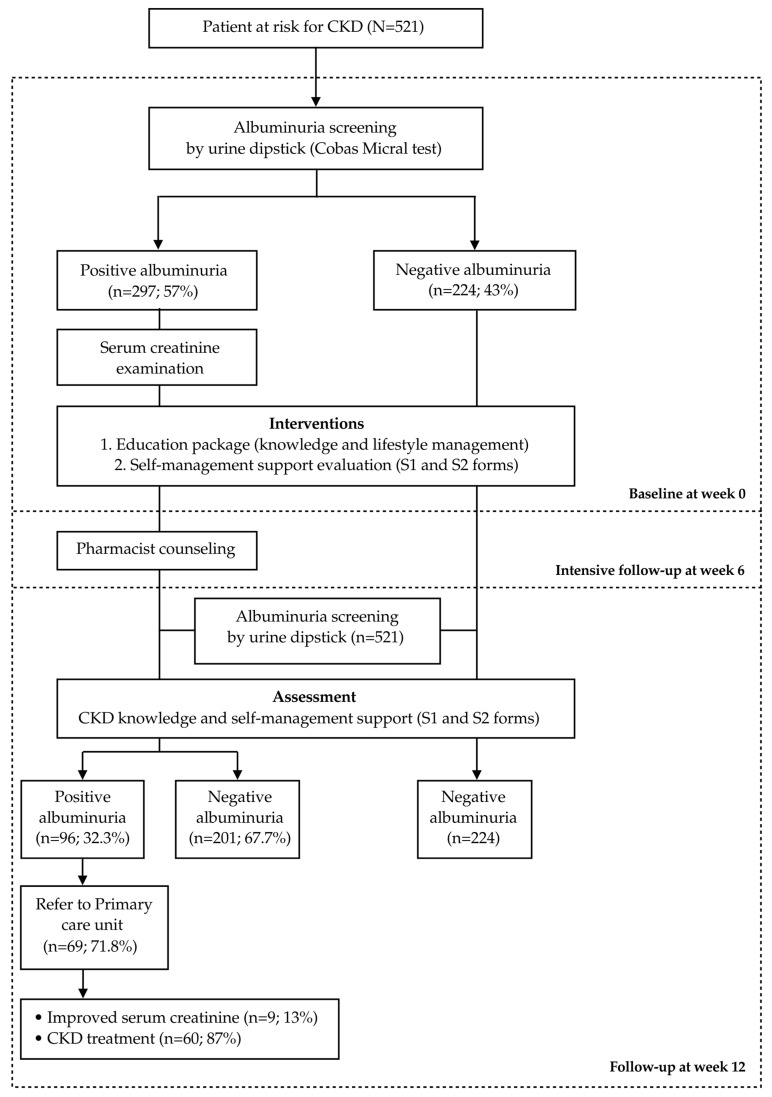
CKD care model for accredited community pharmacies.

In this model, we used albumin urine dipsticks as screening tools in 18 accredited community pharmacies. Urine screening was performed according to Roche company’s instructions. The participants were informed about the CKD video and data sheets. The screening care model is illustrated in Figure 2. At-risk individuals with positive albuminuria upon first-time screening were referred to a partner’s laboratory office to confirm their serum creatinine level. Individuals with positive albuminuria at week 12 were referred to 11 PCUs around the municipal area using a universal pharmacist referral form (PhRF).

### 3.1. Demographics of Participants

The screening results demonstrated that 521 patients were at risk of CKD; 68.7% were female, with a mean age of 54.81 ± 12.11 years. The study participants had a history of taking NSAIDs or nephrotoxic agents (43.6%). Of 521 participants, 297 individuals tested positive for albuminuria at week 0. At week 12, 96 patients tested positive for albuminuria. The demographic data of the participants are shown in Table 2. The characteristics of hypertension, diabetes, or taking nephrotoxic agents, especially NSAIDs, can lead to albuminuria. More than 70% of the positive albuminuria group could not delay kidney progression with lifestyle modification alone. Patients with heart failure, diabetes, and hypertension managed their kidney function through medication, achieving a notable shift to the negative albuminuria group by week 12. Notably, approximately 12% of patients used antihypertensive medications other than ACE inhibitors (ACEIs) or angiotensin receptor blockers (ARBs), 10% used antidiabetic drugs, and 9% used HMG-CoA reductase inhibitors, such as simvastatin or atorvastatin.

### 3.2. CKD Knowledge Score

Most participants were unaware of the role of alcohol consumption and/or smoking. Only 45% were aware of the benefits of weight loss in slowing kidney disease progression, and a few participants were taking diabetes medication. Hypertension is a leading cause of kidney failure. However, after receiving education about CKD from a pharmacist, participants’ self-awareness of CKD improved, with the average score increasing significantly from 6.43 ± 2.34 to 8.06 ± 1.06 (*p* < 0.005) (Table 3).

### 3.3. Self-Management Support (SMS) Score

#### 3.3.1. Self-Management Behavior Assessment (S1) Form

The evaluation of self-management behaviors aimed at mitigating kidney disease progression was conducted using the -S1 form. The study found that the first three low-grade behaviors that contributed to kidney disease progression were consuming low-flavor or bland foods (most patients preferred to eat processed foods), exercising at least 30 min per day or three days per week, and consuming fresh, organic vegetables. All health behavior scores were not different between positive albuminuria or negative albuminuria at week 0. After 12 weeks of follow-up, all study participants scored significantly higher on the behavior assessment (*p* = 0.024) (Table 4). In the negative albuminuria group, the behavior score increased significantly (*p* = 0.042).

#### 3.3.2. Self-Care Ability Assessment (S2) Form

A self-care ability assessment form was also used (S2). To assess the impact of self-care on kidney disease progression, we evaluated participants’ self-care capabilities at baseline and after 12 weeks of receiving guidance on self-care for kidney disease. At baseline, participants rated their self-care capabilities as moderate, with specific areas of focus including chemical food control, food control, eating fresh, non-toxic vegetables, and exercise. After 12 weeks of receiving guidance, participants’ self-care scores increased in all areas (*p* = 0.068) (Table 5).

### 3.4. Logistic Regression Analysis of Urine Albumin Levels

To identify factors associated with urine albumin levels, we employed a univariate analysis and selected variables with a *p*-value < 0.2 for inclusion in the logistic regression analysis. The results of the univariate analysis are presented in Appendix A.

The logistic regression analysis elucidated several significant determinants of positive albuminuria among the study cohort. Participants aged < 60 years demonstrated significantly lower odds of positive albuminuria compared to their counterparts aged ≥ 60 years (OR: 0.44, 95% CI: 0.31–0.72, *p* < 0.005). Clinical conditions, particularly diabetes (OR: 3.69, 95% CI: 2.30–6.03, *p* < 0.005) and hypertension (OR: 2.01, 95% CI: 1.33–3.04, *p* < 0.005), emerged as robust predictors, exhibiting a strong association with increased odds of albuminuria. Conversely, protective factors included a lower BMI (<27.5 kg/m^2^, OR: 0.42, 95% CI: 0.28–0.67, *p* < 0.005), better renal function (eGFR ≥ 60 mL/min/1.73 m^2^, OR: 3.34, 95% CI: 1.16–9.86, *p* = 0.030), lower systolic blood pressure (OR: 0.55, 95% CI: 0.38–0.83, *p* < 0.005), lower diastolic blood pressure (OR: 0.34, 95% CI: 0.23–0.55, *p* < 0.005), and fasting plasma glucose < 126 mmHg (OR: 0.29, 95% CI: 0.19–0.57, *p* < 0.005). Moreover, no current use of pain relievers or NSAIDs (OR: 1.50, 95% CI: 1.41–1.98, *p* = 0.040) and self-management behavior support scores of at least 50 points (OR: 2.04, 95% CI: 1.13–3.82, *p* = 0.020) were significantly correlated with increased odds of albuminuria. However, variables including sex, CKD knowledge, health behavior CKD scores, and MTM did not exhibit statistically significant associations (Table 6).

### 3.5. Sensitivity and Specificity of Urine Dipsticks

Based on Table 7, this study found that the sensitivity and specificity of urine dipsticks were 96.8% and 45.7%, respectively. The positive predictive value (PPV) and negative predictive value (NPV) were calculated as 10.4% and 99.6%, respectively.

## 4. Discussion

This study developed CKD screening and pharmacy self-care support models to help patients at risk of CKD, a criterion selected based on the described guidelines [11,12,13]. These care models aim to slow the progression of CKD to end-stage renal disease (ESRD). However, leakage of other proteins may also be associated with a patient’s condition, which could affect the study. The problems encountered in developing a care program include documents and screening equipment, number of participants, education of pharmacists, and patient follow-up.

Previous studies have screened and referred people at risk of CKD in pharmacies, focusing on people aged 40 years or older who have not been diagnosed with CKD. Using a combination of the Kidney Disease Self-Screening Questionnaire (KIDs) and urine protein screening, 241 patients at risk were identified and referred to PCUs. However, this preliminary study was conducted in only one pharmacy and had limited coordination with cooperative service units [17,18,19]. While the study identified patients with CKD through screening, it also found problems with the referral and tracking of treatment outcomes.

This study was an action research project that aimed to develop a network of screening programs to prevent kidney disease. In collaboration with 13 accredited community pharmacies in the municipal district of Khon Kaen Province, Thailand, this study aimed to provide CKD risk screening and albuminuria testing by community pharmacists. There is evidence of pharmacists successfully providing counseling services to improve patient’s CKD awareness. The results showed that the patient’s satisfaction was high [20].

Screening with urine dipsticks had different sensitivity and specificity values than those reported by the manufacturer. While urine dipstick screening is a useful tool for stimulating patients to modify their behaviors by recognizing their urinary protein leakage, it cannot be used to diagnose CKD. This study is an important contribution to the literature on kidney disease screening, as it raises self-awareness among people at risk. However, it is important to note that the study had a small number of patients who tested positive and were referred to PCUs (n = 69, 71.8%). Additionally, only 13% (n = 9) of the referred group showed an improvement in serum creatinine..

This study, which developed a risk screening protocol for community pharmacies, was an action research study that used both reactive and proactive approaches. Summary and group meetings were necessary to identify problems and improve the guidelines for real-world use. This is consistent with Cha’on et al.’s study [21], which developed guidelines for CKD care in primary care settings. A key consideration for developing successful clinical guidelines is to ensure that they are clear, concise, and easy to implement in real-world settings. The guidelines should also be compatible with existing components of the service and subject to feedback for improvement. For example, the use of a pharmacist referral form (PhRF) has advantages because it provides essential information about the patient, including their insurance status, medical information, reason for referral, and the pharmacist’s contact number. This form is also familiar to medical staff, which can reduce confusion and facilitate the referral process. If the patient does not agree to receive care at the PCU, the pharmacist can continue to follow up on their medication use and recommend behavioral changes at the pharmacy.

The findings of this study reveal that pharmacists regarded the CKD service as efficient, user-friendly, and significantly beneficial for their patients. However, a notable challenge identified was the lack of patient engagement in disease prevention efforts, compounded by a limited understanding of CKD among the population. Furthermore, pharmacists highlighted the importance of interprofessional collaboration, particularly between pharmacists and general practitioners, as a key determinant of the scope and success of pharmacy practice in preventive care. Customer acknowledgment of the pharmacist’s role in disease prevention was also perceived as a critical factor influencing the service’s impact and acceptance [20].

A systematic review examining patient attitudes found that they were more receptive to the availability of medicine-related services than health promotion or screening services, but those who experienced these pharmacy services were highly satisfied with them [22]. In a recent Australian atrial fibrillation screening study, pharmacists perceived combining screening with other established services, such as medication reviews, as an alternative approach to improve service uptake [23]. Similarly, in this qualitative study, pharmacists observed an improvement in the patients’ response to the CKD service when it was integrated with other professional services.

Jane et al. (2019) [24] demonstrated that self-management processes and concepts are effective strategies for empowering individuals with risk factors or chronic diseases to effectively manage their conditions. This study assessed the impact of a self-management intervention against three dimensions: risk factors and prevention, self-management, and outcome. This study found that the intervention was effective for improving short-term outcomes, such as knowledge and behavior changes. However, this study only measured short-term outcomes, and did not assess long-term outcomes, such as health status, quality of life, or healthcare costs. The researchers recommend further studies to assess long-term outcomes [25,26].

The logistic regression analysis identified several factors associated with positive albuminuria. Participants aged < 60 years had lower odds of albuminuria than those ≥ 60 years, consistent with studies linking age-related renal and vascular decline to albuminuria [27,28]. Diabetes and hypertension strongly predicted albuminuria, as these conditions cause microvascular damage and glomerular hyperfiltration [29,30], while lower BMI was protective, aligning with findings that obesity accelerates kidney damage [31]. Better renal function (eGFR ≥ 60 mL/min/1.73 m^2^) reduced albuminuria risk, highlighting the role of preserved filtration capacity [32]. Lower systolic and diastolic blood pressure were protective, underscoring that blood pressure control is important [33]. Elevated fasting plasma glucose increased albuminuria risk, reflecting glycemic control’s critical role in kidney health [34]. Regular NSAID use increased albuminuria odds, consistent with NSAID-induced renal damage [35]. However, the current use of NSAIDs exhibited lower odds; this point is surprisingly opposed to the current knowledge. This might be due to the short-term use of NSAIDs, as the long-term effects may not have been presented.

Participants with higher self-management behavior support scores exhibited increased odds of albuminuria, contrasting findings from previous studies [36,37]. This association may stem from prior participation in CKD health awareness programs provided by public health practitioners, potentially influencing their inclusion in this study and increasing the likelihood of identifying positive albuminuria cases. Variables like sex, CKD knowledge, health behavior scores, and MTM were not significantly associated with albuminuria, reflecting mixed findings in the literature.

We investigated pharmacists’ perspectives on self-management among patients with chronic conditions and explored the feasibility of establishing pharmacist-led self-management initiatives. Our findings suggest that an effective model for chronic disease management should actively involve patients with stable conditions in self-management practices, thereby preventing health deterioration and reducing healthcare costs. Fiona et al. [38] emphasized that the role of pharmacists should extend beyond medication-related responsibilities to encompass broader participation within the primary healthcare system. The previous study suggested that primary care organizations are associated with perceived self-management support. Team-based primary care has been associated with the provision of more patient-centered care [39].

To facilitate the development of pharmacist-led self-management programs, the authors highlighted the importance of government support in expanding pharmacists’ roles in health services. This could be achieved through fostering public–private partnerships with community pharmacists and implementing measures to support this transition. Key recommendations include enabling pharmacists to access electronic health records to enhance the continuity of care and considering the deregulation of certain prescription-only medicines to allow them to be dispensed as pharmacy-only medicines. These measures were identified as critical steps toward gradually establishing pharmacist-led patient self-management within the healthcare system.

This study has several limitations. First, the researchers did not analyze the quality of life of the participants in the screening; therefore, the researchers could not evaluate the relationship of the self-management system to kidney filtration rates and quality of life as part of the study [40]. However, this study found that some topics related to SMS involve avoiding drugs or herbs that affect the kidneys, and non-smoking contributes to a decrease in urine protein levels. Second, this study was conducted in community pharmacies and screened by pharmacists; therefore, we had restrictions on blood sampling, resulting in the need to transfer participants for serum creatinine testing. For further research, screening participants’ blood samples should be arranged in cooperation with network medical or laboratory technicians. Third, the collection of urine samples depended on the participants’ convenience, being affected by the time of day. This may interfere with the results of this study.

## 5. Conclusions

Screening people at risk of kidney disease with microalbuminuria dipsticks can help identify new cases earlier and more frequently, increasing the chances of delaying the progression of CKD. All individuals with positive albuminuria results should be referred to a physician for further evaluation. Community pharmacists can provide patients with counseling on medication use and self-care behaviors. For all individuals with negative albuminuria results, further testing, such as a serum creatinine testing, should be recommended at least annually if they have chronic diseases. A well-planned system for referral and communication can help ensure that patients receive continuous and appropriate care.

## Figures and Tables

**Table 1:** Activities to establish CKD care model in community pharmacies.

Activity	Method	Team Member	Output
Review and update situation	Literature review of knowledge on preventing and slowing down chronic kidney disease	Nephrologist (n = 1) Nurse (n = 2) Community pharmacist (n = 10)	Educational and screening guidance
Design collaboration among accredited community pharmacies	Draft the program to fit with the relevant guidelines and current situation	Community pharmacist (n = 13)	A draft of the collaborative program for CKD care model
Review and draft screening program	Participants’ group discussion (1st)	Community pharmacist (n = 13)	A revised care model for CKD screening
Agreement among team member	Participants’ group discussion (2nd)	Community pharmacist (n = 13)	Establishment of the CKD care model
Work plan	Participants’ group discussion (3rd)	Community pharmacist (n = 13)	Data collection methods and forms related to the outcomes
Training and assessment of screening program	Workshop training	Community pharmacist (n = 13)	Trained team members for the CKD care model

**Table 2:** Demographic data of included participants.

Baseline Characteristics		Number of Participants (%) or Mean ± SD
Gender (N = 521)		
	Female	358 (68.7)
	Male	163 (31.3)
Age (Years)		54.81 ± 2.11
Level of education (N = 521)		
	Primary school	180 (34.6)
	High school/Certificate	101 (19.4)
	Undergraduate	47 (9.0)
	Not defined	193 (37.0)
CKD risks (N = 521) ^a^		
	Diabetes mellitus	121 (23.2)
	Hypertension	135 (25.9)
	Older than 60 years of age	132 (25.3)
	Received NSAIDs or nephrotoxic agents	241 (46.3)
	Family history of CKD	46 (8.8)
	Others	53 (10.2)
Medications (N = 521) ^b^		
	Antihypertension	90 (17.3)
	Antidiabetes	77 (14.8)
	Statins	63 (12.1)
	Antiplatelets	42 (8.1)
	Antidepressants	5 (1.0)
	Alpha-adrenergic blockers	10 (1.9)
	ACEIs/ARBs	54 (10.4)
	Anti-thyroid drugs	6 (1.2)
	NSAIDs	22 (4.2)
	Uric-lowering drugs	7 (1.3)
	Chemotherapy	1 (0.2)
	Iron supplements	65 (12.5)
	Herbal medicines	23 (4.4)
	No medications	306 (58.7)
Urine albumin at baseline (N = 521)		
	Negative (0 mg/dL)	224 (43.0)
	Positive (20, 50, 100 mg/dL)	297 (57.0)

Remark: ^a^ Participants have more than one risk factor. ^b^ Participants have more than one medication.

**Table 3:** CKD knowledge score (N = 521).

CKD Knowledge Score	Week 0 Correct Answer (%)	Week 12 Correct Answer (%)	*p*-Value
1. The kidneys are organs responsible for excreting water and removing waste products from the body.	383 (73.5)	517 (99.2)	0.020
2. The kidneys help balance mineral salts and pH in the body.	322 (61.8)	498 (95.6)	0.017
3. Diabetes and hypertension do not cause kidney disease.	279 (53.6)	476 (91.4)	<0.005
4. People with normal kidney function do not leak protein in their urine and blood waste levels are within normal range.	337 (64.7)	499 (95.8)	<0.005
5. Very salty foods cooked with monosodium glutamate (MSG) cause kidney deterioration faster.	382 (73.3)	513 (98.5)	0.021
6. Drinking alcohol has a reduced effect on kidney function.	243 (46.6)	396 (76.0)	0.006
7. Smoking does not affect kidney function.	172 (33.0)	358 (68.7)	0.007
8. Weight loss can help slow down kidney degeneration.	235 (45.1)	449 (86.2)	<0.005
9. Herbal medicine, Chinese medicine, and herbal pills will help slow down kidney deterioration.	265 (50.9)	455 (87.3)	0.007
10. The use of painkillers or medications does not impair kidney function.	295 (56.6)	450 (86.4)	0.004
11. Taking medication to treat diabetes and high blood pressure causes kidney deterioration.	71 (13.6)	327 (62.8)	<0.005
Total score (11 points) mean ± SD	6.43 ± 2.34	8.06 ± 1.06	<0.005
High (8–11 points)	201 (38.6)	421 (80.8)	<0.005
Medium (4–7 points)	194 (37.2)	99 (19.0)	<0.005
Low (0–3 points)	126 (24.2)	1 (0.2)	<0.005

**Table 4:** Self-management behavior assessment (S1) score (N = 521).

Self-Management Behavior Question	Week 0 (Mean ± SD)	Week 12 (Mean ± SD)	*p*-Value
1. You consume food and beverages that are high in sugar, such as candy, cookies, fruit syrups, smoothies, and soft drinks.	2.82 ± 0.95	2.79 ± 0.99	0.781
2. You eat food that is tasteless or less salty.	2.54 ± 1.03	2.73 ± 0.95	0.032
3. You consume food that has been artificially flavored or processed in a way that alters their original state.	2.87 ± 0.89	3.07 ± 0.91	<0.005
4. You drink more than eight glasses of water each day.	3.14 ± 1.12	3.31 ± 1.03	<0.005
5. You consume fresh, organic, or home-cultivated plant-based food.	2.79 ± 1.09	2.78 ± 1.09	0.990
6. You obtain six-eight hours of sleep per night and experience minimal nocturnal awakenings.	3.24 ± 0.96	3.26 ± 1.03	0.681
7. You are able to effectively cope with and manage stressful situations.	3.16 ± 0.95	3.16 ± 0.92	0.990
8. You incorporate herbal therapies into your medication regimen under the supervision of your healthcare provider.	3.37 ± 1.07	3.52 ± 0.97	0.042
9. You self-medicate without consulting a healthcare professional.	3.38 ± 0.81	3.57 ± 0.78	0.011
10. You engage in moderate-intensity physical activity for at least 30 min per day on most days of the week.	2.58 ± 1.10	2.66 ± 1.23	0.260
11. You are a tobacco user.	3.78 ± 0.75	3.73 ± 0.84	0.831
Total score (44 points)	33.68 ± 4.20	34.58 ± 4.34	0.024
Good level (35–44 points), Number of participants (%)	193 (37.0%)	216 (41.5%)	0.152
Fair level (23–34 points), Number of participants (%)	250 (48.0%)	229 (44.0%)	0.224
Poor level (11–22 points), Number of participants (%)	78 (15.0%)	76 (14.6%)	0.931

**Table 5:** Self-care ability score (N = 521).

Self-Care Ability Question	Week 0 (Mean ± SD)	Week 12 (Mean ± SD)	*p*-Value
1. I am able to manage my intake of sugary food and beverages to protect my kidney health.	6.58 ± 2.44	7.17 ± 2.18	0.004
2. I am able to manage my intake of sodium to protect my kidney health.	6.52 ± 2.53	7.17 ± 2.27	<0.005
3. I am able to control various flavored food for kidney health.	6.98 ± 2.49	7.28 ± 2.27	0.011
4. I maintain adequate hydration to support my kidney health.	7.91 ± 2.28	8.71 ± 1.81	<0.005
5. I consume fresh, organic plant-based food to support my kidney health.	7.02 ± 2.71	7.24 ± 2.74	0.08
6. I obtain 6–8 h of sleep per night to support my kidney health.	7.25 ± 2.52	7.32 ± 2.37	0.667
7. I employ effective stress management strategies to maintain my mental and emotional well-being.	7.52 ± 2.38	7.75 ± 2.36	0.962
8. I avoid medications or herbs that can affect the kidneys.	7.80 ± 2.58	8.54 ± 2.12	<0.005
9. I engage in regular physical activity to support my kidney health.	6.55 ± 2.77	7.47 ± 2.44	<0.005
10. I am a non-smoker.	8.57 ± 2.70	8.85 ± 2.22	0.071
Total score (100 points)	75.84 ± 13.13	77.34 ± 12.72	0.068

**Table 6:** Logistic regression analysis of urine albumin level (N = 521).

Variables		Urine Albumin Level		Adjusted OR	*p*-Value
		Positive Albuminuria	Negative Albuminuria		
Sex					
	Male (n = 163)	101	62	1.29 (0.90–1.92)	0.160
	Female (n = 358)	198	160	1	
Age					
	<60 years (n = 389)	206	183	0.44 (0.31–0.72)	<0.005 *
	≥60 years (n = 132)	93	39	1	
Diabetes					
	Yes (n = 121)	96	25	3.69 (2.30–6.03)	<0.005 *
	No (n = 400)	203	197	1	
Hypertension					
	Yes (n = 135)	94	41	2.01 (1.33–3.04)	<0.005 *
	No (n = 386)	205	181	1	
Systemic infection					
	Yes (n = 2)	2	0	3.69 (0.18–78.28)	0.390
	No (n = 519)	297	222	1	
Regular use of NSAIDs or nephrotoxic medications					
	No (n = 280)	180	100	1.81 (1.38–2.17)	<0.005 *
	Yes (n = 241)	119	122	1	
Decreased renal mass or unilateral kidney, congenital or acquired					
	Yes (n = 2)	2	0	3.72 (0.18–78.28)	0.400
	No (n = 519)	297	222	1	
BMI					
	<27.5 kg/m^2^ (n = 391)	141	178	0.42 (0.28–0.67)	<0.005 *
	≥27.5 kg/m^2^ (n = 121)	78	43	1	
Current use of pain relievers or NSAIDs					
	No (n = 381)	229	152	1.50 (1.41–1.98)	0.040 *
	Yes (n = 140)	70	70	1	
eGFR					
	<60 mL/min/1.73 m^2^ (n = 30)	26	4	3.34 (1.16–9.86)	0.030 *
	≥60 mL/min/1.73 m^2^ (n = 398)	279	145	1	
Systolic blood pressure (SBP)					
	<140 mmHg (n = 291)	149	142	0.55 (0.38–0.83)	<0.005 *
	≥140 mmHg (n = 175)	114	61	1	
Diastolic blood pressure (DBP)					
	<90 mmHg (n = 319)	156	163	0.34 (0.23–0.55)	<0.005 *
	≥90 mmHg (n = 147)	107	40	1	
Fasting plasma glucose (FPG)					
	<126 mg/dL (n = 203)	108	95	0.29 (0.19–0.57)	<0.005 *
	≥126 mg/dL (n = 94)	73	21	1	
Hemoglobin A1C (HbA1C)					
	<7% (n = 17)	14	3	0.52 (0.12–2.33)	0.410
	≥7% (n = 68)	61	7	1	
Health behavior CKD score					
	≥35 points (n = 193)	112	81	1.01 (0.73–1.49)	0.820
	<35 points (n = 328)	187	141	1	
CKD knowledge score					
	≥8 points (n = 201)	114	87	0.91 (0.67–1.37)	0.810
	<8 points (n = 320)	185	135	1	
Self-management behavior support score					
	≥50 points (n = 465)	282	183	2.04 (1.13–3.82)	0.020 *
	<50 points (n = 47)	20	27	1	
MTM ^a^					
	Yes (n = 137)	88	49	0.12 (0.006–1.86)	0.808
	No (n = 8)	8	0	1	

Remark: ^a^ MTM: medication therapy management., * *p*-value < 0.2 for inclusion in the logistic regression analysis.

**Table 7:** Sensitivity and specificity of urine dipsticks.

Screening Results	Characteristics in Population		Total
	CKD	Non-CKD	
Positive albuminuria	31 (TP)	266 (FP)	297
Negative albuminuria	1 (FN)	223 (TN)	224
Total	32	489	521

## Data Availability

The research data are unavailable due to privacy or ethical restrictions.

## Appendix A

### Appendix A.1. Univariate Analysis of Urine Albumin Level

**Variable**	***p*-Value**
Sex	0.005
Age	<0.001
Diabetes	<0.001
Hypertension	<0.001
Systemic infections	0.016
Cardiovascular disease	0.950
Recurrent upper-urinary-tract infections	0.600
Gout or elevated serum uric acid levels	0.449
Regular use of NSAIDs or nephrotoxic medications	<0.001
Decreased renal mass or unilateral kidney, congenital or acquired	0.164
Family history of CKD	0.470
Detected kidney stones or urinary tract stones	0.830
BMI	0.007
Waist circumference (metabolic disease)	0.206
Exercise	0.858
Smoking	0.315
Alcohol consumption	0.546
Unvoid urine	0.883
Drink a small amount of water (<40mL/kg/day)	0.643
Use herbal medicines or traditional medicines	0.329
Current use of pain relievers or NSAIDs	0.074
Current use of dietary supplements	0.325
eGFR	<0.001
Systolic blood pressure (SBP)	0.127
Diastolic blood pressure (DBP)	<0.001
Fasting plasma glucose (FPG)	<0.001
Hemoglobin A1C (HbA1C)	0.086
Serum lipid	0.382
Self-management behavior assessment score (S1)	0.169
CKD knowledge score	<0.001
Self-care ability assessment score (S2)	0.010
MTM	0.005
